# Systematic Bibliometric and Visualized Analysis of Research Hotspots and Trends on the Application of Artificial Intelligence in Ophthalmic Disease Diagnosis

**DOI:** 10.3389/fphar.2022.930520

**Published:** 2022-06-08

**Authors:** Junqiang Zhao, Yi Lu, Shaojun Zhu, Keran Li, Qin Jiang, Weihua Yang

**Affiliations:** ^1^ Department of Nursing, Xinxiang Medical University, Xinxiang, China; ^2^ School of Information Engineering, Huzhou University, Huzhou, China; ^3^ The Laboratory of Artificial Intelligence and Bigdata in Ophthalmology, Affiliated Eye Hospital of Nanjing Medical University, Nanjing, China

**Keywords:** ophthalmic disease, artificial intelligence, diagnosis, bibliometric, CiteSpace

## Abstract

**Background:** Artificial intelligence (AI) has been used in the research of ophthalmic disease diagnosis, and it may have an impact on medical and ophthalmic practice in the future. This study explores the general application and research frontier of artificial intelligence in ophthalmic disease detection.

**Methods:** Citation data were downloaded from the Web of Science Core Collection database to evaluate the extent of the application of Artificial intelligence in ophthalmic disease diagnosis in publications from 1 January 2012, to 31 December 2021. This information was analyzed using CiteSpace.5.8. R3 and Vosviewer.

**Results:** A total of 1,498 publications from 95 areas were examined, of which the United States was determined to be the most influential country in this research field. The largest cluster labeled “Brownian motion” was used prior to the application of AI for ophthalmic diagnosis from 2007 to 2017, and was an active topic during this period. The burst keywords in the period from 2020 to 2021 were system, disease, and model.

**Conclusion:** The focus of artificial intelligence research in ophthalmic disease diagnosis has transitioned from the development of AI algorithms and the analysis of abnormal eye physiological structure to the investigation of more mature ophthalmic disease diagnosis systems. However, there is a need for further studies in ophthalmology and computer engineering.

## Introduction

Artificial intelligence (AI) is a broad term that refers to the use of computers to simulate intelligent behavior with little or no human intervention (Hamet and Tremblay, 2017). It is a multifaceted technology that includes complex algorithms, machine learning, and deep learning, transfer learning, among other components (Balyen and Peto, 2019; Kora et al., 2022). Medicine has long been identified as one of the most promising fields for the application of AI. Many clinical decision support systems have been proposed and developed by researchers since the mid-twentieth century (Miller, 1994). AI has been used in ophthalmology to diagnose diseases in conjunction with imaging technologies such as optical coherence tomography, and fundus fluorescein angiography (Ruiz Hidalgo et al., 2017; Hemalakshmi et al., 2020; Wan et al., 2021c; Ran et al., 2021). In addition, several simple and low-cost diagnostic system models have been under development (Bourouis et al., 2014; Metha et al., 2021). As a possible solution for the screening of major ophthalmic diseases and telemedicine, AI has been applied to the study of ophthalmic disease diagnosis. For example, diabetic retinopathy, glaucoma, hypertensive retinopathy, high myopia, age-related macular degeneration, familial amyloidosis, cataract, and other related conditions have been investigated using this technology (Fang et al., 2017; Asaoka et al., 2019; Araujo et al., 2020; Juneja et al., 2020; Kessel et al., 2020; Zhou et al., 2020; Wan et al., 2021a; Grzybowski and Brona, 2021; Szeskin et al., 2021; Xu et al., 2022). AI may have an impact on medical and ophthalmic practice in the coming decades, based on the results of several published reports (Ting et al., 2019; Dai et al., 2021).

Previous studies have used bibliometric methods to study the application of artificial intelligence in ophthalmic diseases in China (Dong et al., 2021; Koh et al., 2021; Saeed et al., 2021; Boudry et al., 2022). However, there is no bibliometric research on the application of artificial intelligence in ophthalmic disease diagnosis. This study aimed to gain a comprehensive understanding of the general use and research frontier of artificial intelligence in ocular illness detection by examining multiple aspects. For example, Scientific Citation Index (SCI) papers on the application of artificial intelligence to ocular illness diagnosis were analyzed using bibliometric approaches. The analysis emphasized data related to countries, regions, institutions, journals, research categories, keywords, and references. A critical aspect of our study was the development of a repeatable and unbiased strategy for exploring the active knowledge frontier in the research field. In particular, we examined the active areas of applied artificial intelligence, future development areas, and potential hurdles, relative to ocular disease diagnostics. This report is intended to serve as a resource for artificial intelligence professionals, ophthalmologists, diagnosticians, and medical imaging researchers.

## Materials and Methods

**FIGURE 1:**
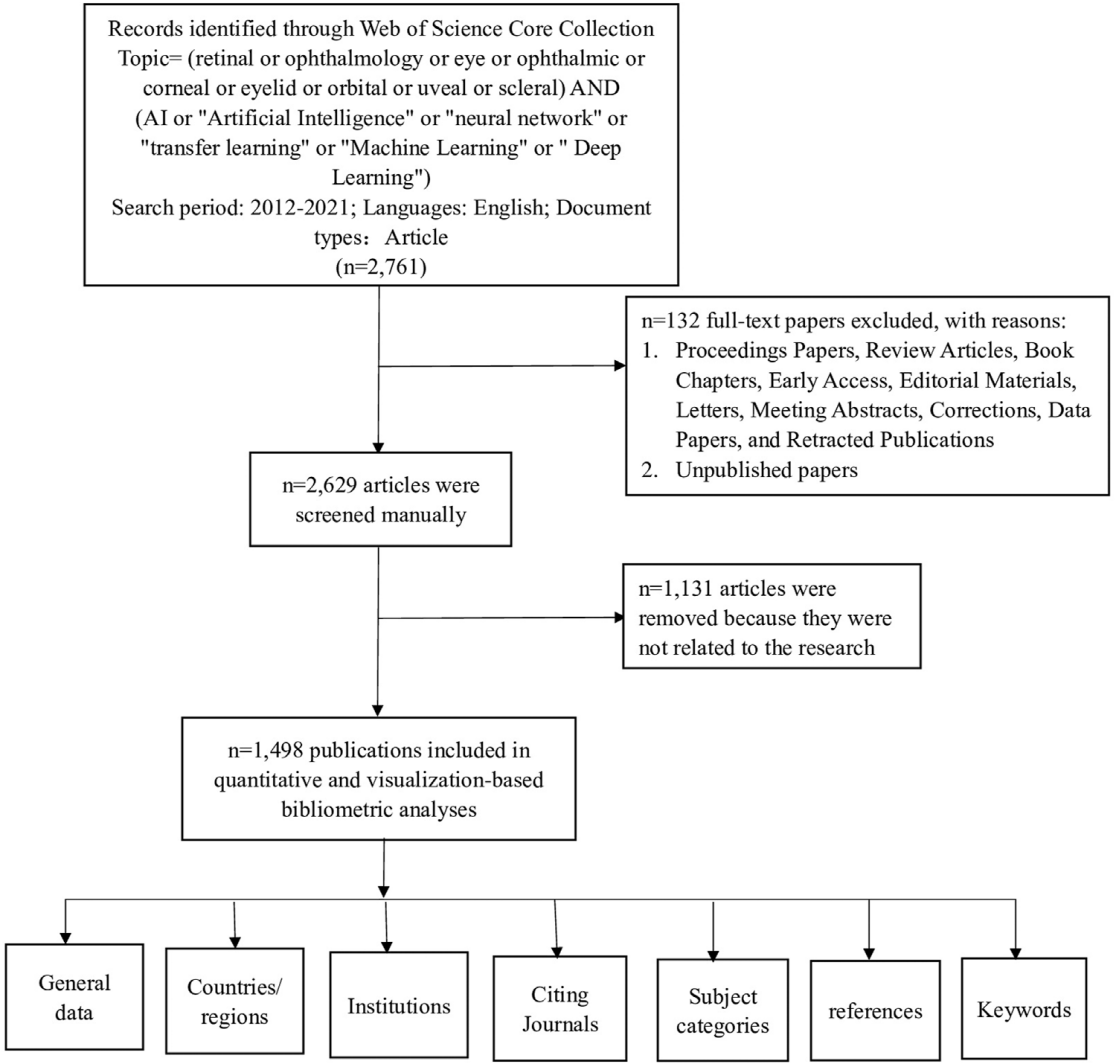
Frame flow diagram showing the detailed selection criteria and bibliometric analysis steps for the study of the application of AI in the diagnosis of ophthalmic diseases.

On 1 April 2022, citation data published between 1 January 2012, to 31 December 2021, were retrieved from the Web of Science Core Collection (WoSCC). These data were independently verified by two authors (Weihua Yang and Yi Lu). The search formula was TS= (retinal or ophthalmology or eye or ophthalmic or corneal or eyelid or orbital or uveal or scleral) AND (AI or “Artificial Intelligence” or “neural network” or “transfer learning” or “Machine Learning” or “Deep Learning”) AND (diagnos* or grad* or classification). The search selected English literature and articles and excluded early access, proceedings papers, book chapters, data papers, and retracted publications. To obtain the most accurate analysis results, we manually deleted data after reading the title and abstract of each literature. The criteria for manual exclusion are as follows: 1) the research discipline does not include medicine; 2) The study organ is an organ other than the eye; 3) The research method does not use artificial intelligence method; 4) The study disease is not an eye disease. All the data we included in the analysis were the research of artificial intelligence in the diagnosis of ophthalmic diseases. For each publication, we extracted the title, publication year, country or region, institution, journal, references, and keywords. The detailed search and analysis processes are depicted in Figure 1.

Collaborative networks of countries, institutions, journals, keywords, references, and research categories were analyzed using CiteSpace.5.8. R3 and Vosviewer. The article describes all citation features.

## Results

### Distribution of Articles by Publication Year

**FIGURE 2:**
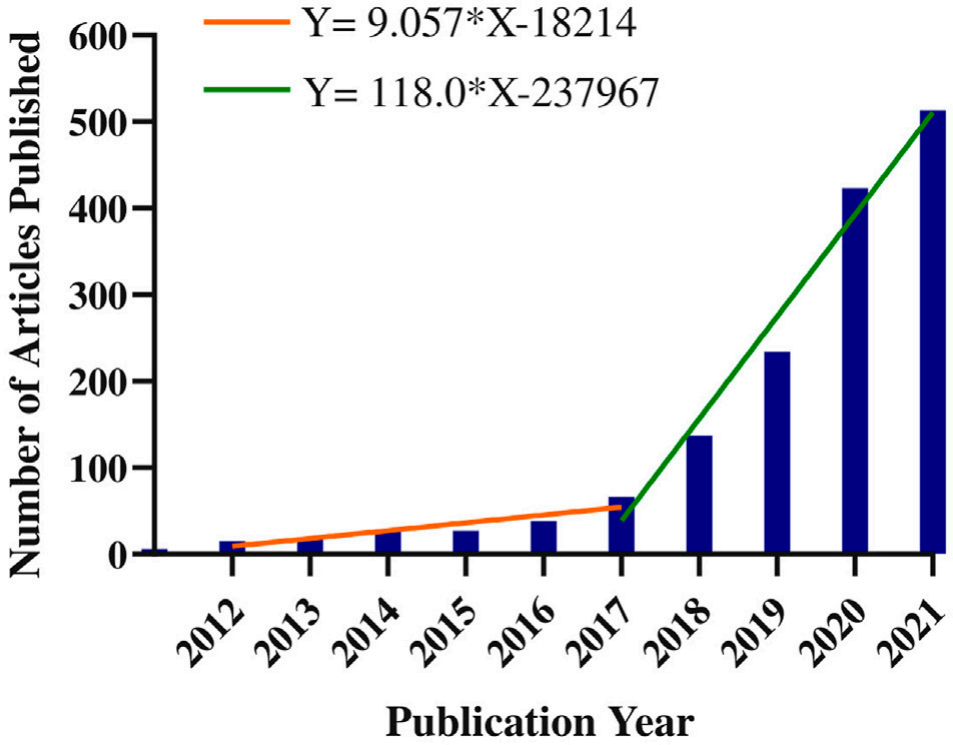
Annual number of publications on the application of AI in the diagnosis of ophthalmic diseases from 2012 to 2021.

This study analyzed 1,498 papers that were published between 2012 and 2021 that focused on the use of AI in the diagnosis of ophthalmic diseases. Using the Web of Science (WoS) citation analyzer to count the annual number of citations retrieved and using the duplicate removal function of CiteSpace software to verify the data of the number of citations. The number of annually published reports for this period is shown in Figure 2. Since 2018, the annual number of articles on the application of AI in the diagnosis of ophthalmic diseases has exceeded 100 and has increased rapidly in subsequent years.

### Countries or Regions

**FIGURE 3:**
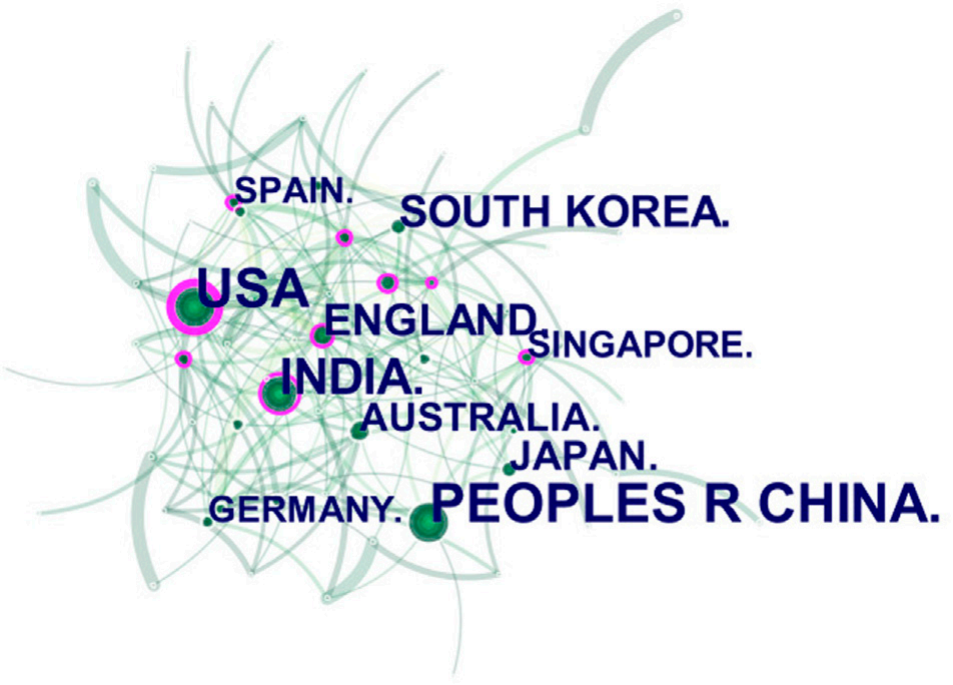
Cooperation of countries or regions that contributed to publications on the use of AI for the diagnosis of ophthalmic diseases from 2012 to 2021.

The citation analyzer of WoS database is used to count the number of documents sent by countries or regions, and the default setting of CiteSpace software analyzes the cooperative relationship between countries and regions. These citations involve a total of 95 countries or regions. The size of each label and green node area in Figure 3 represents the number of documents sent. Countries with large green node areas include the people’s Republic of China (415 articles), the United States (365 articles) and India (263 articles). The connection between nodes represents the cooperative relationship between regions. Countries with more connecting lines have regions with strong influence, The area of purple circle indicates the influence of national documents, which is expressed by the centrality in Table 1. The purple circle in the United States has the largest area (0.25), indicating that the articles published in the United States in the field of ophthalmic diagnosis have the greatest overall influence. The data in Table 1 objectively substantiate these conclusions. The H index can accurately reflect academic achievement (Hirsch, 2005). The higher the centrality and H index, the greater the influence of a paper. In general, China had the largest number of articles and the United States had the greatest influence.

**TABLE 1:** Top 10 countries or regions with publications on the application of the use of AI in the diagnosis of ophthalmic diseases from 2012 to 2021.

Rank	Countries or regions	Counts	Centrality	H-index
1	China	415	0.07	38
2	United States	365	0.25	48
3	India	263	0.12	31
4	England	119	0.18	24
5	South Korea	109	0.04	21
6	Japan	76	0.03	19
7	Australia	73	0.08	19
8	Singapore	53	0.14	20
9	Germany	48	0.04	15
10	Spain	48	0.17	13

### Institutions

**FIGURE 4:**
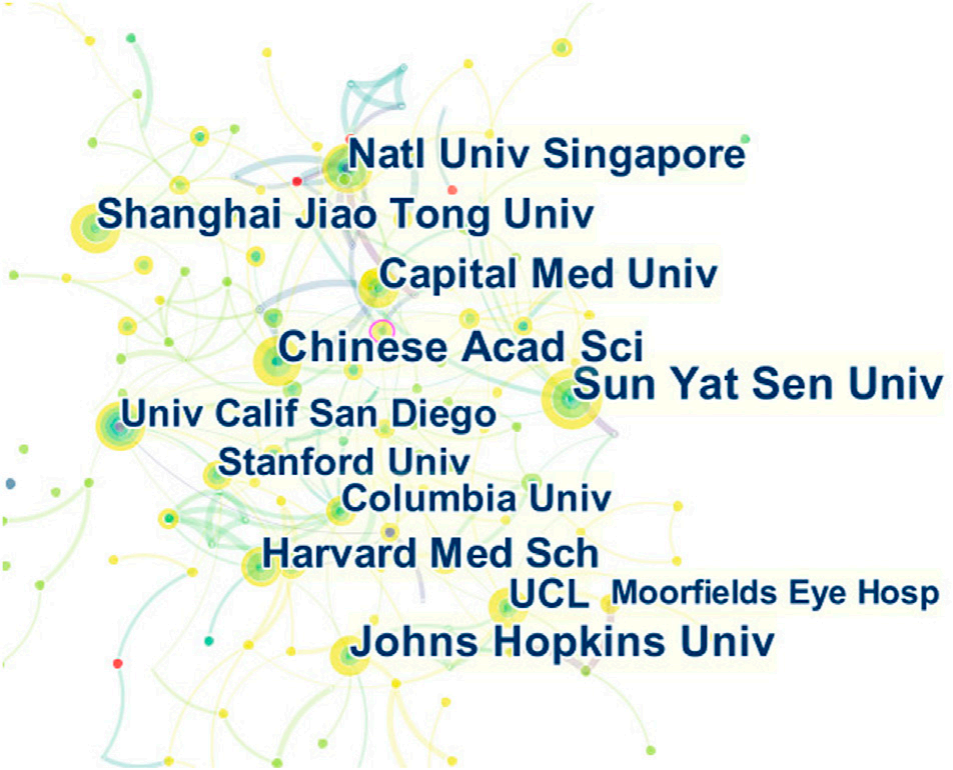
Cooperation of institutions that contributed to publications on the use of AI in the diagnosis of ophthalmic diseases from 2012 to 2021.

Table 2 lists the top 10 institutions for published articles that were analysed. The displayed data is outcome from the default settings of CiteSpace software and vosviewer software. These included three American institutions, three England institutions, three Chinese institutions, and one Singapore institution. Two of the top five institutions with the highest h-index were from the United States and England. The connection between the tags in Figure 4 shows the inter-agency cooperation. The node size indicates the number of documents sent.

**TABLE 2:** Top 10 Institutions with publications on the application or the use of AI in the diagnosis of ophthalmic diseases from 2012 to 2021.

Rank	Institution	Count	H-index	Countries or regions
1	University of California System	49	17	United States
2	Harvard University	47	15	United States
3	University of London	46	13	England
4	University College London	44	13	England
5	Moorfields Eye Hospital NHS foundation Trust	40	12	England
6	Chinese Academy of Sciences	38	10	Chinese
7	Sun Yat Sen University	38	10	Chinese
8	National Univesity of Singapore	35	17	Singapore
9	Johns Hopkins University	33	12	US
10	Shanghai Jiao Tong University	31	8	Chinese

#### Journals and Research Category

**FIGURE 5:**
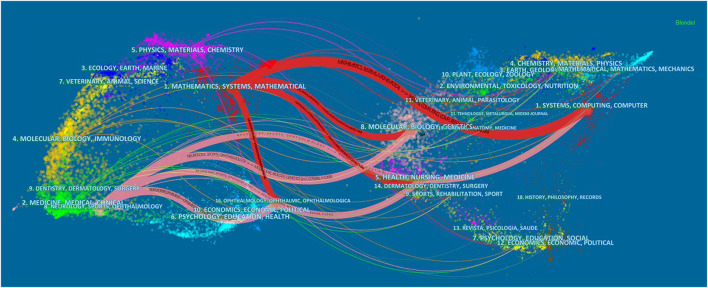
Dual map overlay of journals that contributed to publications on the use of AI in the diagnosis of ophthalmic diseases from 2012 to 2021.

The documents in the cited journals constitute the knowledge base of the referenced articles. The research fields in highly cited journals constitute an active area of interest or hotspot. We use CiteSpace to draw the citation relationship in the field of journal research. The paths of the two colors shown in Figure 5 represent the citation relationship of highly active research fields. Among them, the red path represents the classification of the journals with the most papers. The research field of the citing journals is represented on the left, and the research field of the cited journals is shown on the right. The knowledge-based research fields of AI application in ophthalmic disease diagnosis in the recent 10 years include systems/computing/computer/molecular/biology/genetics/health/nursing/medicine/ophthalmology, which constitute the hotspot subjects involved in the research frontier such as mathematics/systems/mathematical/neurology/sports/ophthalmology. Tables 3, 4 list the discipline categories of the citing journals and cited journals/proceedings that rank among the top ten in terms of citations. The most common research field of the citing journals includes engineering technology/computers. The discipline that was most involved in the extracted version was classified as medicine/ophthalmology.

**TABLE 3:** Top 10 citing journals of publications on the use of AI in the diagnosis of ophthalmic diseases from 2012 to 2021.

Rank	Citing journals	Research fields	Counts	Journal impact factor 2020
1	IEEE Access	Engineering Technology/Computer: Information System	73	3.367
2	Translational Vision Science & Technology	Medicine Ophthalmology	68	3.283
3	Scientific Reports	Comprehensive journal	59	4.38
4	Biomedical Signal Processing and Control	Engineering Technology/Engineering: Biomedicine	37	3.88
5	Computer Methods and Programs in Biomedicine	Engineering Technology/Computer: Interdisciplinary Applications	33	5.428
6	Multimedia Tools and Applications	Engineering Technology/Computer: Information System	32	2.757
7	American Journal of Ophthalmology	Medicine/Ophthalmology	30	5.258
8	PLOS ONE	Comprehensive journal	30	3.24
9	IEEE Transactions on Medical Imaging	Medicine/Computer: Interdisciplinary Applications	27	10.048
10	Neurocomputing	Engineering Technology/Computer: Artificial Intelligence	24	5.719

**TABLE 4:** Top 10 cited journals of publications on the use of AI in the diagnosis of ophthalmic diseases from 2012 to 2021.

Rank	Citing journals	Research fields	Counts	Journal impact factor 2020
1	Ophthalmology	Medicine/Ophthalmology	702	12.079
2	Investigative Ophthalmology & Visual Science	Medicine/Ophthalmology	660	4.799
3	IEEE Transactions on Medical Imaging	Medicine/Computer: Interdisciplinary Applications	579	10.048
4	British Journal of Ophthalmology	Medicine/Ophthalmology	527	4.638
5	American Journal of Ophthalmology	Medicine/Ophthalmology	437	5.258
6	PLOS ONE	Comprehensive journal	430	3.24
7	Journal of Perianesthesia Nursing	Engineering Technology/Computer: Artificial Intelligence	396	1.084
8	JAMA-Journal of the American Medical Association	Medicine/Internal Medicine	384	56.274
9	Computers in Biology and Medicine	Engineering Technology/Biology	342	4.5892
10	IEEE Transactions on Pattern Analysis and Machine Intelligence	Engineering Technology/Computer: Artificial Intelligence	338	16.389

#### Keywords

**FIGURE 6:**
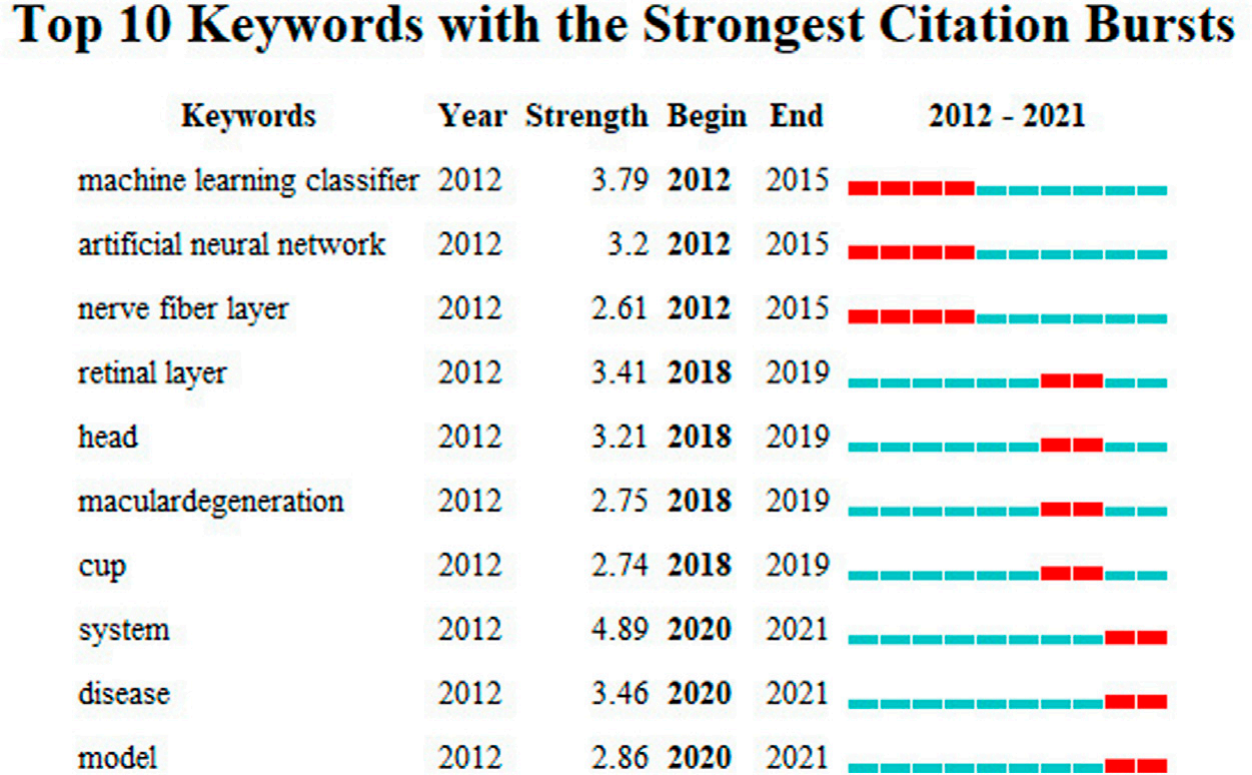
Keywords with the strongest citation bursts for publications on the use of AI in the diagnosis of ophthalmic diseases from 2012 to 2021.

To better understand the adoption of AI in the field of ophthalmic disease diagnosis in the recent 10 years based on an analysis diagram of keyword co-occurrence cooperation network, the emerging keywords that progressed over time were analyzed. This represented the migration of research hotspots. The default setting of CiteSpace is changed to the following mode: “Year Per Slice” = 2, “Top N%" = 30.0%, and “Minimum Duration” = 1. We get the result of Figure 6. The red square in Figure 6 represents emerging keywords for the investigated timeline. The bursts keywords from 2012 to 2021 included machine learning classifier (2012–2015), artificial neural network (2012–2015), nerve fiber layer (2012–2015), retinal layer (2018–2019), head (2018–2019), macular degeneration (2018–2019), cup (2018–2019), system (2020–2021), disease (2020–2021), and model (2020–2021).

#### Citing Articles and References

**FIGURE 7:**
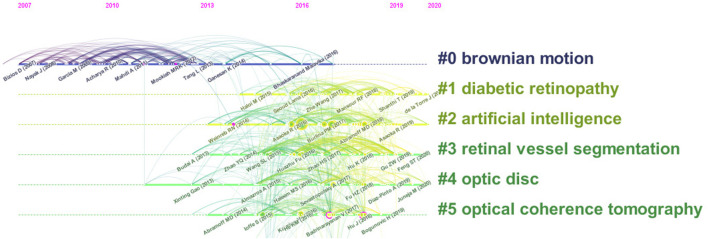
Co-cited reference timeline map of publications on the use of AI in the diagnosis of ophthalmic diseases from 2012 to 2021.

The cited documents were highly related to the research topics. Using the default setting of CiteSpace to cluster the co cited documents and choose label clusters with indexing terms. The cluster labels that represented the research frontiers of the co-cited documents were obtained from these documents. The cited literature constituted the knowledge base of the research, and the size of the clusters obtained from the literature are listed in order from top to bottom on the right side of Figure 7. The largest cluster label #0“Brownian motion” was obtained during the pre-period of the application of AI for ophthalmic diagnosis, which was 2007–2017, and was a research hotspot. Table 5 lists the top ten citing literature from “times cited in all databases “among the relevant literature based on the application of AI for ophthalmic diagnosis. It was determined that AI techniques are promising for use in the diagnosis of ophthalmic diseases although there are some limitations associated with their use.

**TABLE 5:** Top 10 citing articles on the application of the use of AI in the diagnosis of ophthalmic diseases from 2012 to 2021.

Rank	Title of citing documents	DOI	Times cited	Interpretation of the findings	Research limitations
1	Development and Validation of a Deep Learning Algorithm for Detection of Diabetic Retinopathy in Retinal Fundus Photographs (Gulshan et al., 2016)	10.1001/jama.2016.17216	2,591	Deep machine learning-based algorithms have high sensitivity and specificity for detecting actionable diabetic retinopathy.	1. Image subtly was difficult for ophthalmologists to interpret.
					2. The algorithm only displayed the lesion grade and did not count the actual diabetic retinopathy lesions.
					3. Ophthalmic examination image data sets were limited in number.
					4. The algorithm identified only diabetic retinopathy and diabetic macular edema.
					5. The clinical utility of user interface settings is unknown.
2	Development and Validation of a Deep Learning System for Diabetic Retinopathy and Related Eye Diseases Using Retinal Images From Multiethnic Populations With Diabetes (Ting et al., 2017)	10.1001/jama.2017.18152	769	Deep learning systems for the evaluation of retinal images in multiethnic diabetic patients are highly sensitive and specific for identifying diabetic retinopathy and associated eye diseases.	1. Inconsistencies in diagnostic criteria among ophthalmologists.
					2. The algorithm only displayed the lesion grade and did not count the actual diabetic retinopathy lesions.
					3. Diagnosis of all diabetic macular edema still requires the use of optical coherence tomography
3	Segmenting Retinal Blood Vessels with Deep Neural Networks (Liskowski and Krawiec, 2016)	10.1109/TMI.2016.2546227	481	Deep neural networks are a viable methodology for medical imaging.	Only a limited set of image data including drive database, start database, and chase database, were used. These data sets contained limited examination populations.
4	Automated Identification of Diabetic Retinopathy using Deep Learning (Gargeya and Leng, 2017)	10.1016/j.ophtha.2017.02.008	478	This study presented a novel deep learning-based automatic feature learning method for Diabetic Retinopathy detection that offered an efficient, low-cost, and objective diagnostic method, which has high efficiency without relying on clinicians to manually review and grade images.	1. It was difficult for the algorithm to automatically distinguish between partial and early-stage cases of diabetic retinopathy.
					2. Limitations in the number of image datasets analyzed.
5	Improved Automated Detection of Diabetic Retinopathy on a Publicly Available Dataset Through Integration of Deep Learning (Abramoff et al., 2016)	10.1167/iovs.16-19964	403	Deep learning enhanced algorithms have the potential to improve the efficiency of diabetic retinopathy screening	1. The ophthalmic disease examination images in the disclosed data set represented only part of the clinical examination images.
					2. Different reference standards may cause differences in the performance of device measurement algorithms.
					3. The approach lacked the same flexibility as an actual clinical diagnosis.
6	Pivotal Trial of an Autonomous AI-Based Diagnostic System for Detection of Diabetic Retinopathy in Primary Care Offices (Abramoff et al., 2018)	10.1038/s41746-018-0040-6	355	The algorithm developed in this study is the first autonomous artificial intelligence diagnosis system for the detection of diabetic retinopathy in any medical field authorized by the United States Food and Drug Administration.	1. Limitations of the spectrum of disease tested in the system.
					2. The sensitivity of the AI system was lower than that of a similar AI system that was tested using a laboratory dataset.
7	A Cross-Modality Learning Approach for Vessel Segmentation in Retinal Images (Li et al., 2016)	10.1109/TMI.2015.2457891	330	A novel supervised vascular segmentation method for retinal images was presented, which has potential applications in retinal image diagnostic systems	1. There are specific requirements for the quality of the images to be diagnosed.
					2. Special algorithms that simultaneously predict all pixel labels in one retinal image block remain unknown.
8	Automatic Segmentation of Nine Retinal Layer Boundaries in OCT Images of Non-Exudative AMD Patients using Deep Learning and Graph Search (Fang et al., 2017)	10.1364/BOE.8.002732	274	A new framework combining convolutional neural network and pattern search method was proposed for automatic segmentation of nine-layer boundaries of retinal optical coherence tomography image	The framework was validated in only subjects with non-exclusive age-related macular degeneration.
9	Joint Optic Disc and Cup Segmentation Based on Multi-Label Deep Network and Polar Transformation (Fu et al., 2018)	10.1109/TMI.2018.2791488	277	This study proposed a deep learning architecture called M-net, which jointly solved the problem of the optic disc and cup segmentation in fundus images in a single-stage multi-label system, and developed a function for glaucoma screening	The image data sets selected for verification were limited and included only ORIGA and SCES datasets.
10	Efficacy of a Deep Learning System for Detecting Glaucomatous Optic Neuropathy Based on Color Fundus Photographs (Li et al., 2018)	10.1016/j.ophtha.2018.01.023	272	This study proposed a deep learning system for detecting referable glaucomatous optic neuropathy with high sensitivity and specificity.	The ophthalmic images used in the study were only collected from Chinese hospitals, resulting in limitations associated with the image data

## Discussion

### Principal Results

Based on the preceding results, it is apparent that the published literature on the application of AI in the diagnosis of ophthalmic diseases has increased sharply in the past 5 years. This indicates that this research field has gradually attracted significant interest in recent years. The World Economic 2016 Forum identified the open artificial intelligence ecosystem as one of the ten most important emerging technologies (President et al., 2016). Gulshan, V et al. established that deep learning algorithms had high sensitivity and specificity for detecting diabetic retinopathy and macular edema (Gulshan et al., 2016). Although it is impossible to predict the number of literature on AI for ophthalmic disease diagnosis that will be published in the future, it is still a promising research field and the efficacy of the application of AI has been confirmed by many studies (Giardini and Livingstone, 2020).

In terms of the number of national documents, the United States has the highest centrality and h-index. This indicates that this country has a leading position in this research field. In addition, developed countries such as Britain, Spain, and Singapore exhibited strong centrality and influence. Although China published a large number of papers, it lacked highly cited articles. The top five research institutions in terms of the number of publications are in the United States and the United Kingdom. From the perspective of the h-index, the national unity of Singapore had a strong impact. This was observed in the analysis results for national document issuance. From the analysis of the research field of the journal, it is evident that in recent years, research focused on the use of computer engineering technology combined with a knowledge base of ophthalmology to develop more suitable ophthalmic disease detection systems. AI is widely used to identify ophthalmic diseases, which is typically based on the analysis of ophthalmic images (Xu et al., 2021a; Wan et al., 2021b; Xu et al., 2021b). In addition, this research also includes the detection of genes related to ophthalmic diseases (Saikia and Nirmala, 2022), ocular metabolites (Myer et al., 2020), and pathology ocular metabolites (Nezu et al., 2020). Areas of active interest and the research frontiers of AI in ophthalmic disease diagnosis can be identified based on the clustering timeline of emerging keywords and co-cited references. The titles and abstracts of 936 articles published in 2020 and 2021 were examined.

Table 6 lists the top ten diseases in the recent 2 years, among it we can see that the hottest disease with the ophthalmic diagnosis using AI technology is “diabetic retinopathy”. A classic study in 2016 was that Gulshan, V and others developed a deep machine learning algorithm based on 128,175 retinal images, which has high sensitivity and specificity in detecting diabetes retinopathy (Gulshan et al., 2016). In 2017, Ting, DSW and others obtained high sensitivity and specificity when using the deep learning system to evaluate the diagnostic images of patients with diabetes retinopathy and related eye diseases from multiple ethnic groups (Ting et al., 2017). Li, SC and others pointed out that the conditions for using artificial intelligence system to replace ophthalmologists are not mature (Li et al., 2022). Therefore, using AI technology to diagnose diabetes retinopathy or other ophthalmic diseases requires more research.

**TABLE 6:** Top ten diseases mentioned in the published literature from 2020 to 2021.

Rank	Disease	Counts	Rank	Disease	Counts
1	Diabetic Retinopathy	340	6	Cataract	54
2	Glaucoma	294	7	Retinopathy of Prematurity	35
3	Age-related Macular Degeneration	128	8	Ophthalmic Tumor	31
4	Corneal Disease	71	9	Myopia	31
5	Diabetic Macular Edema	64	10	Intraocular Pressure	26

### Hot Knowledge Base in the Start Period

According to the results of the cluster analysis of CO cited references, it is not difficult to determine during the early stage of the sharp increase in the number of studies, the most important knowledge base is “Brownian motion.”

Previous studies have shown that using fractional Brownian motion to model medical examination images can provide better global texture indicators than traditional texture feature-based measurement methods (Mcgarry and Deriche, 1997). Early detection of glaucoma is important in the prevention of blindness. Yun, WL et al. used digital fundus images to extract texture features based on fractal dimension and Brownian motion. Specifically, they performed two-dimensional two-level discrete wavelet transform on the images, extracted energy and entropy data, and finally developed a highly specific and sensitive early glaucoma diagnosis model (Yun et al., 2014b). They also proposed a final stage of automatic detection of diabetic retinopathy using Brownian motion characteristics, namely, proliferative diabetic retinopathy (Yun et al., 2014a). Earlier literature also confirmed that the Brownian motion model can be used to classify normal and abnormal ultrasound liver images (Wu et al., 1992).

### Research Hotspots

The research hotspots can be identified based on an analysis of emerging keywords. The emergent keywords in different periods represent different research hotspots.

The emerging keywords from 2012 to 2015 were “Machine Learning Classifier,” “artificial neural network,” and “nerve fiber layer.” This suggests that the performance of different intelligent algorithms was under investigation for application to ophthalmic disease diagnosis. A subfield of artificial intelligence technology is machine learning. It employs algorithms in a systematic manner to synthesize the potential relationship between data and information (Awad and Khanna, 2015). Andersson, S et al. used the visual field print output of 99 glaucoma patients and 66 healthy people to compare the sensitivity and specificity of the results obtained for a glaucoma diagnosis system based on artificial neural network and direct diagnosis by ophthalmologists. This study confirmed that an artificial neural network has higher specificity and sensitivity and fewer classification errors compared to doctors (Andersson et al., 2013). Yousefi, S et al. compared the detection of glaucoma progression using different machine learning classifiers based on longitudinal structure data sequences extracted from retinal nerve fiber layer thickness measurement and visual function data obtained from standard automatic visual field examination and evaluated the performance of these classifiers (Yousefi et al., 2014). To improve the effectiveness of treating open-angle glaucoma, Ein Oh et al. investigated the application of a screening method to distinguish open-angle glaucoma from suspected glaucoma without visual field testing. They used five open-angle glaucoma risk prediction models that were created based on 8,958 subjects (including patients with suspected open-angle glaucoma) using an artificial neural network. It was established that the artificial neural network method was a cost-effective screening tool for distinguishing between patients with open-angle glaucoma and glaucoma suspect subjects (Oh et al., 2015).

The emerging keywords from 2018 to 2019 were “retinal layer,” “head,” “macular degeneration,” and “cup.” This indicates that the research focus transitioned to the study of various ophthalmic diseases and anatomical structures. Color fundus photography facilitates the examination of the optic disc to determine the cup-to-disc ratio, which is important in the diagnosis of glaucoma. Al-Bander et al. proposed a new method based on deep learning, which used the combination of a convolution network and DenseNet to segment optical discs and optical cups. Four data sets for image detection were then used to evaluate its effectiveness (Al-Bander et al., 2018). Although the image quality of optical coherence tomography (OCT) needs to be improved and the scanning duration leads to patient discomfort, this imaging modality has become an established clinical routine for *in vivo* imaging of optic nerve head tissue, which is very important in the diagnosis of various ophthalmic diseases (Du et al., 2014). Devalla, SK et al. developed a customized deep learning method to remove the noise in a single frame OCT B-scan. The network proposed in this study performs denoising in less than 20 milliseconds (Devalla et al., 2019). Based on the analysis of chorioretinal OCT images, clinicians and researchers have a better understanding of the diagnosis of a series of ophthalmic diseases under different conditions (Bussel et al., 2014). In practical applications, choroidal boundary segmentation usually requires manual segmentation, which is time-consuming. Kugelman, J et al. proposed several depth learning methods based on complete convolution to accurately determine the location of the choroidal boundary of interest. Artificial intelligence technology was compared with manual boundary segmentation and standard image analysis technology as part of the study. Furthermore, the investigation established the advantage of deep learning methods in chorioretinal boundary analysis and the segmentation of OCT images (Kugelman et al., 2019).

The emerging keywords from 2020 to 2021 were “system,” “disease,” and “model,” which indicate that researchers have begun to develop various systematic diagnostic models for the study of ophthalmic diseases. The proportion of doctors and patients in China is unbalanced, and the regional distribution of medical capital is uneven (National Health Commission, 2020). To minimize these biases, Zheng, B et al. designed five intelligent models for the diagnosis of fundus diseases using transfer learning. These models can detect normal eyes and four common fundus diseases including retinal vein occlusion, high myopia, glaucoma, and diabetic retinopathy (Zheng et al., 2021). However, the limitation of this study is that the models can only diagnose four common fundus diseases, which may be misdiagnosed in the case of other ophthalmic diseases. Ahn, H used machine-learning artificial intelligence to develop a model to classify the severity of emerging ophthalmic diseases, with an accuracy of nearly 100% (Ahn, 2020). The authors reported that the relative lack of data set samples was a major limitation of the study. To prevent vision loss caused by corneal diseases and to improve the early diagnosis of corneal diseases, Elsawy, A et al. proposed a deep learning network based on corneal OCT images, Fuchs’ cochlear dystrophy, and keratoconus. The developed algorithm was then used to evaluate the data set of 16721 OCT images. It was determined that the algorithm was superior to other network learning procedures (Elsawy and Abdel-Mottaleb, 2021).

As the adoption of artificial intelligence increases and the technology is continually applied to a wide array of medical fields, more intelligent detection methods will be required for ophthalmic diseases diagnosis.

### Limitations in Citing Articles

By summarizing the limitations of the top ten cited literature, it was determined that the constraints of AI in ophthalmic disease diagnosis can be divided into the following five categories: 1) The clinical examination standard is highly subjective, which leads to difficulties in the design of intelligent auxiliary diagnosis system; 2) Limited detectability of ophthalmic diseases in intelligent auxiliary diagnosis systems; 3) The data set used in most training models included a limited examination population; 4) The diagnosis of artificial intelligence of ophthalmic diseases is still in the stage of auxiliary diagnosis, and requires the use of traditional clinical examination tools; 5) The scope limitations of validating intelligent auxiliary diagnosis systems.

To develop a more robust and usable diagnostic system, it is necessary to obtain more types and larger data sets. For example, the collection of ophthalmic examination data from different races, countries, or regions (Bellemo et al., 2019; Raumviboonsuk et al., 2019; Al Turk et al., 2020). More disease types should also be included in these studies, such as pterygium, familial amyloidosis, and thyroid-associated ophthalmopathy (Kessel et al., 2020; Zamani et al., 2020; Xu W. et al., 2021; Song et al., 2021). In addition, more ophthalmologists with different levels of training should participate in the screening stage of the data set and the examination stage of the algorithm to obtain clinically-based diagnoses. Finally, by increasing the number of clinical cases of the validation system and utilizing statistical approaches to achieve high sensitivity and specificity, the utilization rate of artificial intelligence models can be further improved.

## Conclusion

In summary, the training of intelligent algorithms based on the analysis of images is of growing interest. There is ongoing worldwide research on the use of AI in ophthalmic diagnosis. In particular, the United States is the most influential country in this research field. The application of artificial intelligence technology to ophthalmic diagnosis has revolutionized the clinical landscape of ophthalmologists and patients. This technology facilitates more accurate diagnosis and remote diagnosis services. However, there are limitations associated with these approaches. For example, the credibility of the training model is questionable and is often not recognized by institutions for practical clinical work, even if it has been shown to have high sensitivity and specificity. In addition, contemporary research is focused on several relatively well-known diseases, whereas the number of studies on other diseases is small, and a mature diagnostic system has not been developed. At present, the research focus has shifted from the development of artificial intelligence algorithms and the analysis of the abnormal ocular physiological structure to research on ophthalmic disease diagnosis systems. To address the existing limitations, it is necessary to obtain more national and ethnic ophthalmic data to train and test the algorithm. This is a huge task. In addition to computer engineering experts who primarily develop algorithms, ophthalmologists from different regions and with different levels of experience need to participate in this endeavor.

## Data Availability

The original contributions presented in the study are included in the article/Supplementary Material, further inquiries can be directed to the corresponding authors.
